# A Case of Membraneous Croup Relieved by Tracheotomy

**Published:** 1873-10-15

**Authors:** H. Coakley

**Affiliations:** Manitowoc, Wis.


### A CASE OF MEMBRANEOUS CROUP RELIEVED BY TRACHEOTOMY.

BY H. COAKLEY, M.D., MANITOWOC, WIS.

On the 4th I was called to see a boy who, as his father stated, was complaining of a bad cold, which he had taken some seven days before, and had been getting worse gradually ever since. This being in the evening, and not having time to go and see him then, I gave medicine as indicated by symptoms as stated by the father, promising to call next morning.

5th.—Called to see him, and found to my surprise that he was suffering from membraneous croup (the symptoms and history as given by the mother readily confirmed). I made a pharyngeal examination, and found the throat in a healthy state; and, not having the proper instruments at hand, was obliged to forego an examination of the larynx, to which the symptoms pointed as the seat of the trouble. The symptoms were so bad that I had but little hope of being able to accomplish any good by the ordinary course of treatment. However, I prescribed the ordinary treatment as advised by Profs. Flint, Davis, and Hartshorn; but the agent that I had ordered and directed to have been used, and in which I had the most confidence, viz.: the inhalation of the steam from pouring water on fresh lime, was not used at all, as they stated the boy (five years old) could not live under such treatment. Called next morning, 6th, and found him alive, and that was about all. Told the parents, in my judgment, he could not last many hours, and the only hope was in an operation. Called in Drs. Ritchard and Simons, who agreed with me, and advised an operation; so, with the consent of the parents, I began the operation without the use of an anæsthetic, as we thought his low state would not permit of it. Having made the necessary opening down to the trachea with but little hæmorrhage, although I met with a troublesome plexus of veins (middle thyroid artery ligated), one of which had to ligate, and washing until all bleeding had stopped, made an opening of about three-quarters of an inch in the trachea with prompt relief to the patient. In absence of silver tube, used large-sized catheter (silver), but its presence produced so much irritation that I was obliged to take it out. There was much plastic deposit thrown out, one part of which measured nearly six inches. Although the struggles of the child made the cutting very tedious, I found the most trouble was before me, viz.: to keep the wound open. Having nothing better at hand, I took a long, slender ear forceps and connected that, the spring of which was strong enough to keep the edges of the wound apart, and not only that, but I found it very convenient to draw out the plastic matter as it presented at the opening. As an antiseptic, I used the carbolized spray, which appeared to answer all requirements. I would say that I tried the silver wire to keep the edges of the tracheal wound apart, but the force of coughing tore them out. In connection with the local treatment I gave calomel, pot nit., and, after the inflammatory stage was passed, tonics, with beef essence and all the milk he would drink. Kept a thin gauze over the wound. Sixth day withdrew the forceps, and by the ninth or tenth the wound was closed by granulation, and discharged but very little.

